# β-catenin/cyclin D1 mediated development of suture mesenchyme in calvarial morphogenesis

**DOI:** 10.1186/1471-213X-10-116

**Published:** 2010-11-26

**Authors:** Anthony J Mirando, Takamitsu Maruyama, Jiang Fu, Hsiao-Man Ivy Yu, Wei Hsu

**Affiliations:** 1 Department of Biomedical Genetics, Center for Oral Biology, James P Wilmot Cancer Center, University of Rochester Medical Center, 601 Elmwood Avenue, Box 611, Rochester, NY 14642, USA

## Abstract

**Background:**

Mouse genetic study has demonstrated that Axin2 is essential for calvarial development and disease. Haploid deficiency of β-catenin alleviates the calvarial phenotype caused by Axin2 deficiency. This loss-of-function study provides evidence for the requirement of β-catenin in exerting the downstream effects of Axin2.

**Results:**

Here we utilize a gain-of-function analysis to further assess the role of β-catenin. A transgenic expression system permitting conditional activation of β-catenin in a spatiotemporal specific manner has been developed. Aberrant stimulation of β-catenin leads to increases in expansion of skeletogenic precursors and the enhancement of bone ossification reminiscent to the loss of Axin2. The constitutively active signal promotes specification of osteoprogenitors, but prevents their maturation into terminally differentiated osteoblasts, along the osteoblast lineage. However, the prevention does not interfere with bone synthesis, suggesting that mineralization occurs without the presence of mature osteoblasts. β-catenin signaling apparently plays a key role in suture development through modulation of calvarial morphogenetic signaling pathways. Furthermore, genetic inactivation of the β-catenin transcriptional target, cyclin D1, impairs expansion of the skeletogenic precursors contributing to deficiencies in calvarial ossification. There is a specific requirement for cyclin D1 in populating osteoprogenitor cell types at various developmental stages.

**Conclusion:**

These findings advance our knowledge base of Wnt signaling in calvarial morphogenesis, suggesting a key regulatory pathway of Axin2/β-catenin/cyclin D1 in development of the suture mesenchyme.

## Background

The mammalian skull consists of neurocranium and viscerocranium that are formed from skeletogenic mesenchyme derived from both mesoderm and neural crest [1]. The cranial skeletogenic mesenchyme mainly undergoes intramembranous ossification to form the bone plates [2]. This process differs from endochondral ossification in the appendicular and axial skeletons, which prior formation of cartilage templates is required. The growth of calvaria is then able to accommodate expansion of the brain [2]. During calvarial morphogenesis, cranial sutures serve as skeletogenic growth centers where undifferentiated stem cells develop into calvarial osteoprogenitors, which differentiate to become pre-osteoblasts/osteoblasts localized to the osteogenic front and periosteum. The mature cells then deposit and mineralize bone matrix. Osteoblasts either die by apoptosis or are embedded in the matrix, becoming osteocytes.

A patent suture is necessary for continuous growth of the skull bones. Defects in either cell proliferation, differentiation, or apoptosis affecting the process of intramembranous ossification have been shown to induce premature fusion of cranial sutures, leading to development of craniosynostosis [3]. Abnormalities in the developing suture cause severe malformations of the skull, leading to disruption of brain development. Cranial dysmorphism resulting from suture closure defects can be familial or sporadic in origin [3-5]. Although linkage analyses have shown that genetic mutations are associated with synostosis-related syndromes, the mechanism underlying suture development remains largely elusive. It remains a challenge to understand the maintenance of suture patency which may require undifferentiated cells to be present. These naïve cells are thought to be localized in the suture mesenchyme. Better understanding of the genetic regulatory network conveying signals to orchestrate the suture morphogenetic processes promises important insights into pathogenesis of congenital deformities.

Our previous work has linked an evolutionary conserved Wnt signal transduction pathway for the first time to craniosynostosis [6]. Mice with genetic inactivation of Axin2, a negative regulator targeting β-catenin degradation, exhibit suture abnormalities. The synostosis phenotype is caused by accelerated intramembranous ossification mediated through a dual role of β-catenin in both the expansion of osteoprogenitors and the maturation of osteoblasts [6,7]. Moreover, haploid deficiency of β-catenin alleviates the premature closure and calvarial osteoblast defects. The loss-of-function analysis thus suggests the requirement of β-catenin in Axin2 mediated calvarial morphogenesis.

In this study, we employ a gain-of-function analysis to determine if aberrant activation of β-catenin induces calvarial defects reminiscent to the Axin2 mutant. A transgenic system integrating the tetracycline-dependent activation and Cre-mediated recombination methods has been developed. Our findings demonstrate that inducible stimulation of β-catenin causes severe suture abnormalities resembling those with the Axin2 ablation. The expansion of skeletogenic precursors and their subsequent differentiation are altered, leading to increased bone ossification. β-catenin signaling plays a critical role in development of the suture mesenchyme through modulation of calvarial morphogenetic signaling pathways. Furthermore, analyzing mice with disruption of the β-catenin transcriptional target, cyclin D1, we reveal its essential role in the expansion of skeletal precursors. Cyclin D1 is required for propagation of specific types of osteoprogenitors at various developmental stages.

## Results

**Figure 1:**
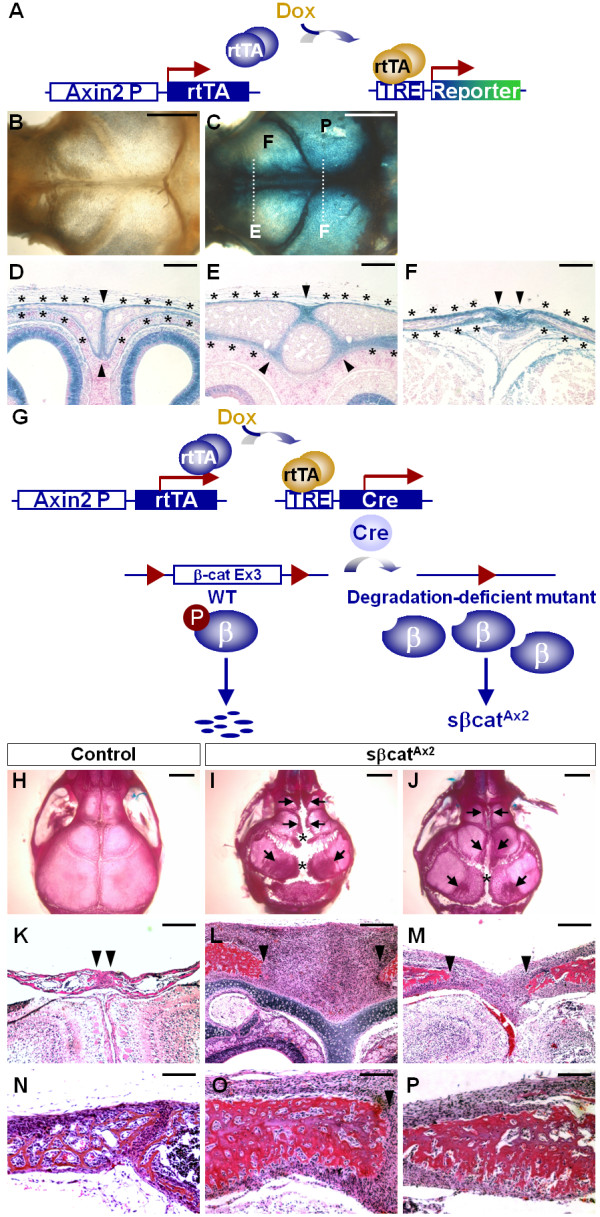
**Inducible activation of β-catenin causes deficiencies in calvarial morphogenesis**. (A) A schematic represents the strategy for targeted expression in the Axin2-expressing cells. Four day-old mice carrying Axin2-rtTA and TRE-lacZ transgenes without (B) or with (C) the Dox treatment, starting at E16.5, were analyzed for expression of the reporter. β-gal staining reveals strong expression of lacZ in the developing skull. Sections of the β-gal stained skulls at three different levels (D, internasal suture; E, metopic suture; F, sagittal suture) indicated in C exhibit targeted gene expression in the suture mesenchyme, osteogenic fronts (arrowheads), and periosteum (asterisks). (G) A scheme represents the development of a transgenic system, which integrates the tetracycline-dependent activation and Cre-mediated recombination, to express a degradation-deficient form of β-catenin (sβ-cat) in the Axin2-expressing cells. (H-J) Skeletal staining of control (H, genotype: Axin2-rtTA; β-cateninΔEx3Fx/+) and sβcat^Ax2 ^(I, J) mice reveals skull abnormalities caused by the expression of sβ-cat around P17. (K-P) H&E staining shows that conditional expression of sβ-cat expands metopic suture mesenchyme (K-M) and enhances bone ossification (N-P). The suture regions are enlarged in the sβcat^Ax2 ^mutants (L, M) compared to the control (K). Frontal bone volumes are increased in the sβcat^Ax2 ^skulls (O, P) compared to the control (N). Arrowheads indicate the osteogenic fronts. F, frontal bone; P, parietal bone. Scale bars, 2 mm (B, C, H-J); 200 μm (D-F, K-M); 100 μm (N-P).

We previously showed that Axin2 is expressed in the suture mesenchyme, osteogenic fronts and periosteum essential for suture morphogenesis [6]. To investigate if stimulation of β-catenin leads to a causal effect on osteoblast proliferation and differentiation similar to the Axin2 mutant phenotype, a transgenic system permitting manipulation of gene activity in the Axin2-expressing cells was created. Using the Axin2-rtTA transgenic mouse strain expressing an improved version of rtTA, rtTA2^S^-M2, under control of the Axin2 promoter, we tested the capability of conditional gene expression in the developing calvaria. Mice carrying the Axin2-rtTA transgene were crossed with TRE-lacZ mice to obtain double transgenic mice (Figure 1A). Their mother was treated with doxycycline (Dox) to induce expression of the lacZ reporter in the 4 day-old pups. The double transgenic skull exhibited strong β-gal staining while no signal was detected in the control (Figure 1B, C). Compared with the endogenous Axin2 expression pattern that is detected by β-gal staining using the Axin2^lacZ ^knock-in mouse strain [6], the results showed that expression of the lacZ reporter was successfully targeted to the suture mesenchyme, osteogenic fronts and periosteum of developing sutures (Figure 1D-F). Similar results could also be obtained using a different reporter, TRE-H2BGFP, suggesting that the Axin2-rtTA transgenic strain permits inducible gene expression during calvarial morphogenesis [8].

We then developed a transgenic system combining the tetracycline-dependent activation and Cre-mediated recombination methods to determine if aberrant activation of β-catenin affects suture development (Figure 1G). This advanced system permits inducible stimulation of β-catenin during calvarial morphogenesis. It allows us to test if constitutive activation of β-catenin signaling causes suture abnormalities, leading to defects in skeletogenesis, similar to that observed in the Axin2 nulls. First, mice carrying the Axin2-rtTA transgene were crossed with the TRE-Cre mice to obtain the Axin2^Cre ^model. Next, the β-catΔEx3Fx allele was introduced to the Axin2^Cre ^mice. In these triple transgenic animals, Cre recombinase was produced in the Axin2-expressing cells in the presence of Dox. The expressed Cre then deleted exon 3 of β-catenin to generate a truncated protein. As a result, a stabilized dominant protein (sβ-cat), which lacks important phosphorylation sites [9] and cannot be targeted for proteosome degradation, was generated in the Axin2-expressing cells (Figure 1G). This sβcat^Ax2 ^mouse model permits stimulation of β-catenin in a spatiotemporal specific fashion during formation of the craniofacial skeleton. Inducible expression of sβ-cat in the Axin2-expressing cells had significant effects on craniofacial skeletogenesis (Figure 1H-J). The suture regions of the sβcat^Ax2 ^mice were drastically expanded (Figure 1K-M). In addition, bone ossification was enhanced in these mutants compared to the control (Figure 1N-P).

**Figure 2:**
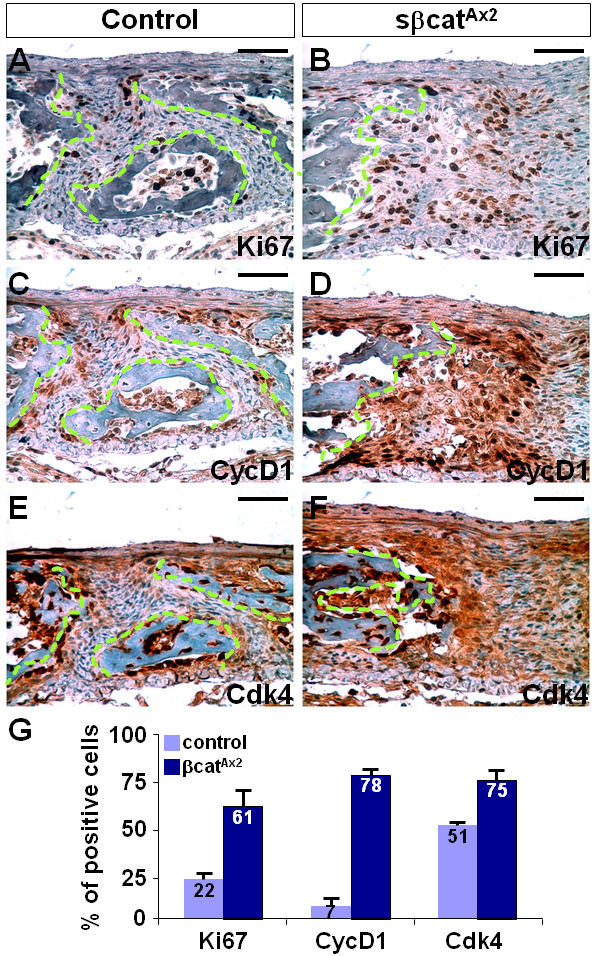
**Stimulation of β-catenin interferes with cell proliferation in the developing suture mesenchyme**. (A, B) Expression of sβ-cat increases the number of Ki67 positive cells suggesting that proliferation of skeletal precursors is enhanced in the metopic suture at one week after birth. The numbers of cyclin D1 (CycD1; C, D) and Cdk4 (E, F) positive cells also increase drastically in the mutants. The broken lines define the suture region. (G) Graph indicates the average percentage of the positive stained cells in the controls and mutants (mean + SEM). Scale bars, 50 μm (A-F).

Further investigations revealed an increased number of Ki67 positive cells in the sβcat^Ax2 ^suture region (Figure 2A, B, G; *p *value = 0.0024, n = 4). Similar to that observed in the Axin2 nulls, the expression of a direct Wnt target, cyclin D1, was highly stimulated (Figure 2C, D, G; *p *value = 0.0026, n = 3). This was accompanied by stabilization of its target Cdk4, which promotes cell cycle G1/S transition (Figure 2E, F, G; *p *value = 0.0425, n = 3). Statistical analysis showed that these alterations in cell proliferation significantly contribute to the mesenchymal defects of the sβcat^Ax2 ^sutures (Figure 2G). Constitutive stimulation of β-catenin causes a detrimental effect on the expansion of skeletal precursors in the developing suture.

**Figure 3:**
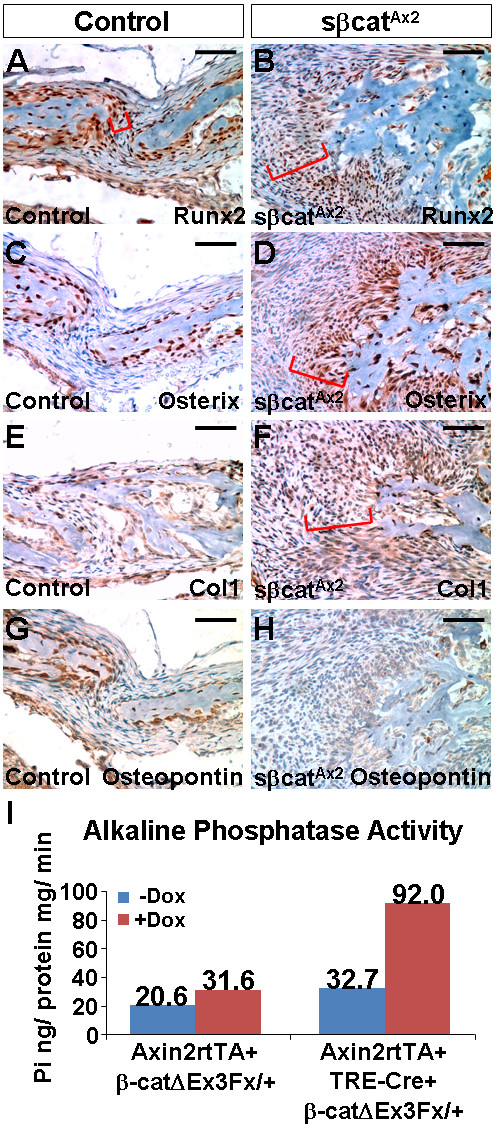
**Development of the osteoblast lineage is affected in the sβcat^Ax2 ^mice**. Immunostaining analysis examines the expression of osteogenic markers, Runx2 (A, B), Osterix (C, D), Col1 (E, F) and Osteopontin (G, H), in the one week old control (A, C, E, G) and sβcat^Ax2 ^(B, D, F, H) mice. Brackets indicate the expression domains. (I) The expression of sβ-cat accelerates osteoblast differentiation in vitro. The control (Axin2rtTA; β-cateninΔEx3Fx/+) and mutant (Axin2rtTA+; TRE-Cre+; β-cateninΔEx3Fx/+) calvarial cells, isolated from the newborn skulls, were induced for differentiation for 6 days. Alkaline phosphatase liquid assay shows that the presence of Dox has minimal effect on osteoblast differentiation in the controls. However, the inducible expression of sβ-cat results in accelerated differentiation. The diagram is a representative of two independent experiments. Scale bars, 50 μm (A-H).

We next determined if osteoblast lineage development is affected in the sβcat^Ax2 ^mutants. Osteogenic markers expressed at different stages along the osteoblast differentiation pathway were examined (Figure 3). At the osteogenic fronts, we detected that the area of Runx2-expressing cells is expanded in the mutants (Figure 3A, B). This is also accompanied by expansion of the regions containing Osterix and Col1 positive cells (Figure 3C-F). In contrast, cells expressing high levels of Osteopontin, a late marker for mature osteoblasts, were missing at the sβcat^Ax2 ^osteogenic fronts with only very low levels of Osteopontin detected compared to the control counterparts (Figure 3G, H). This suggests that terminal differentiation of mature osteoblasts expressing high levels of Osteopontin is prohibited. Nonetheless, there is no question that bone ossification is aberrantly elevated in the mutant skulls (Figure 1). To test if inducible expression of sβ-cat promotes differentiation of calvarial precursors into osteoblasts, we then used an ex vivo culture system. In the control culture, which lacks the TRE-Cre transgene to delete β-catenin exon3, no significant effect on osteoblast differentiation was observed in the presence of Dox (Figure 3I). However, the Dox-mediated expression of sβ-cat caused a ~3 fold induction of alkaline phosphatase activity, suggesting that the differentiation process was stimulated (Figure 3I).

**Figure 4:**
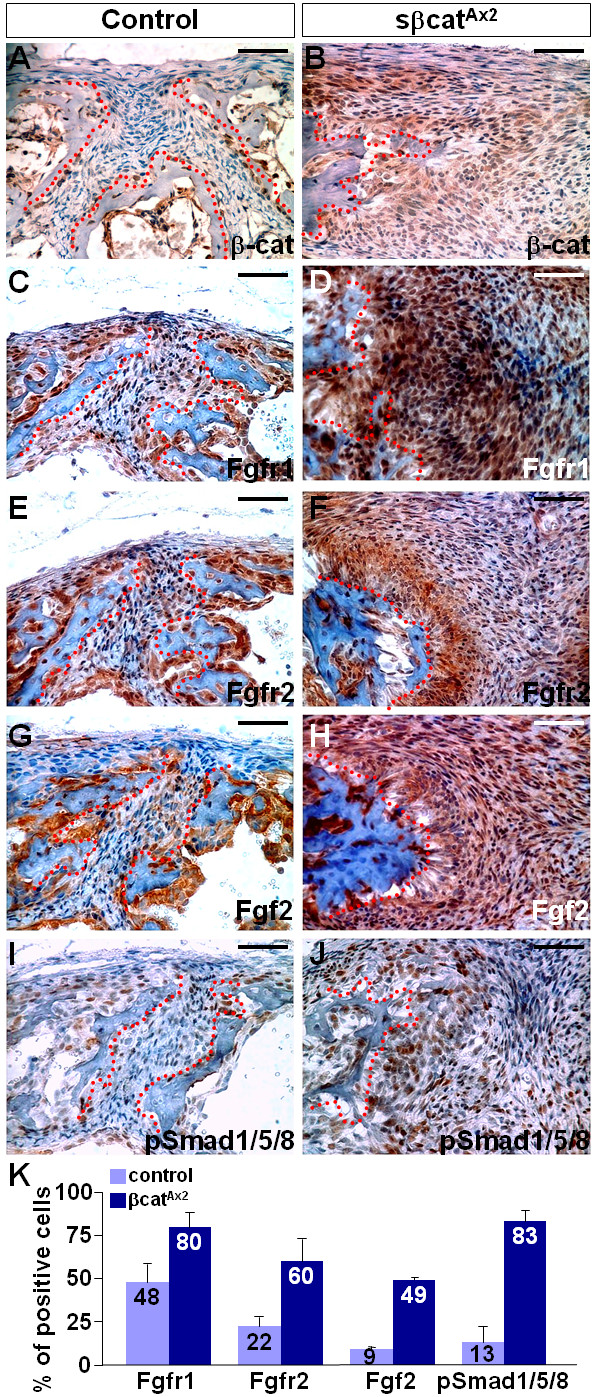
**FGF and BMP signaling is stimulated by sβ-cat in the developing suture**. Immunostaining analysis reveals that β-catenin gain-of-function mutation alters the population of the β-catenin (A, B), Fgfr1 (C, D), Fgfr2 (E, F), Fgf2 (G, H) and phosphorylated Smad1/5/8 (I, J) expressing cells in the metopic suture at one week after birth. The broken lines define the suture region. (K) Graph shows the average percentage of positive stained cells in the controls and mutants (mean + SEM). Scale bars, 50 μm (A-J).

The loss of Axin2 elevates the signaling activities of FGF and BMP pathways critical for calvarial development and disease [6,7]. Therefore, we examined whether these calvarial morphogenetic pathways are interfered by the expression of the dominant β-catenin protein in a manner similar to the Axin2 mutants. Immunostaining analysis with antibodies recognizing the activated/non phosphorylated form of β-catenin [10] confirmed its elevated levels in the sβcat^Ax2 ^suture (Figure 4A, B). Fgfr1, Fgfr2 and Fgf2, expressed in the suture mesenchyme are important regulators for bone ossification and maintenance of suture patency. Deregulation of these molecules has been extensively linked to development of synostosis-related syndromes [3-5,11,12]. FGF signaling, including Fgfr1 (Figure 4C, D, K; *p *value = 0.015, n = 3), Fgfr2 (Figure 4E, F, K; *p *value = 0.0173, n = 3) and Fgf2 (Figure 4G, H, K; *p *value = 0.0092, n = 3), affected by Axin2 ablation [6,7], is also highly active in the sβcat^Ax2 ^mutants. Stimulated expression of phosphorylated Smad1/5/8 proteins found in the Axin2 nulls was also observed by the expression of sβ-cat (Figure 4I, J, K; *p *value = 0.0033, n = 3). The results imply that β-catenin stimulation is able to recapitulate the effect of the Axin2 ablation, leading to activation of the FGF and BMP pathways (Figure 4K).

Our findings demonstrated that stimulation of β-catenin in the skeletogenic mesenchyme promotes expansion of skeletal precursors, specification of osteoblast cell types, maturation of matrix-producing osteoblasts, and FGF and BMP signaling. These phenotypic and regulatory defects, reminiscent to the Axin2 deficiency, thus provide evidence to support an essential role of β-catenin in mediating the Axin2-null phenotypes using a gain of function approach.

**Figure 5:**
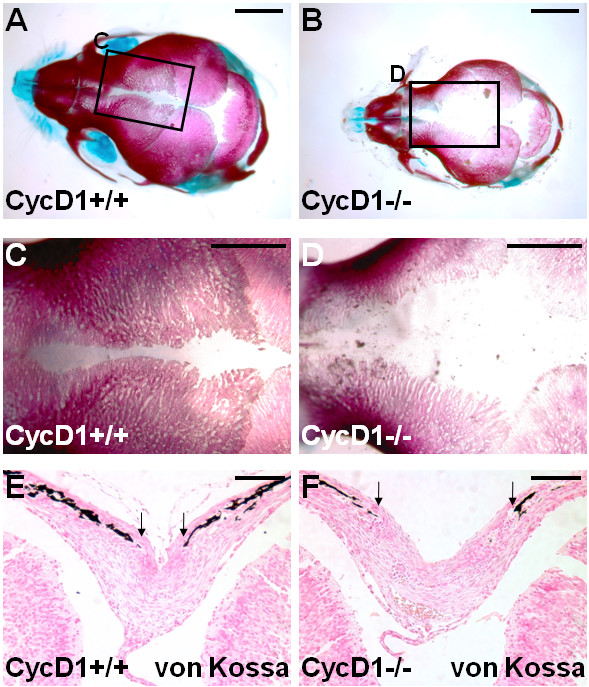
**Cyclin D1 is essential for skull and suture development**. (A-D) Skeletal staining of P0 mice reveals calvarial ossification defects associated with inactivation of cyclin D1 (CycD1+/+; A, C and CycD1-/-; B, D). Enlargements of insets in A and B are shown in panels C and D. (E, F) The loss of cyclin D1 affects ossification of the bone plate analyzed by von Kossa staining. Arrows indicate the osteogenic fronts of CycD1+/+ (E) and CycD1-/- (F). Scale bars, 2 mm (A, B); 1 mm (C, D); 100 μm (E, F).

Disruption of Axin2/β-catenin signaling consistently alters the regulation of its downstream transcription target, cyclin D1, in the canonical Wnt pathway [6,7]. The number of cyclin D1 positive cells also increases from 7% in the control to 78% in the sβcat^Ax2 ^suture region (Figure 2G). However, there is a lack of knowledge on the essential role of cyclin D1 in calvarial morphogenesis, contributing to the defects caused by aberrant Axin2/β-catenin signaling. To definitively assess the requirement of cyclin D1 in development of the craniofacial skeleton, we examined the skull defects caused by its disruption in mice. The loss of cyclin D1 apparently interfered with bone mineralization causing a delay of ossification at the newborn stage (Figure 5A, B). A large foramen was observed in the cyclin D1-null skulls, especially in the frontal bones (Figure 5C, D). Von Kossa staining showed decreased mineralization, resulting in a wider suture region, caused by the mutation (Figure 5E, F). Cyclin D1 is therefore essential for suture morphogenesis.

**Figure 6:**
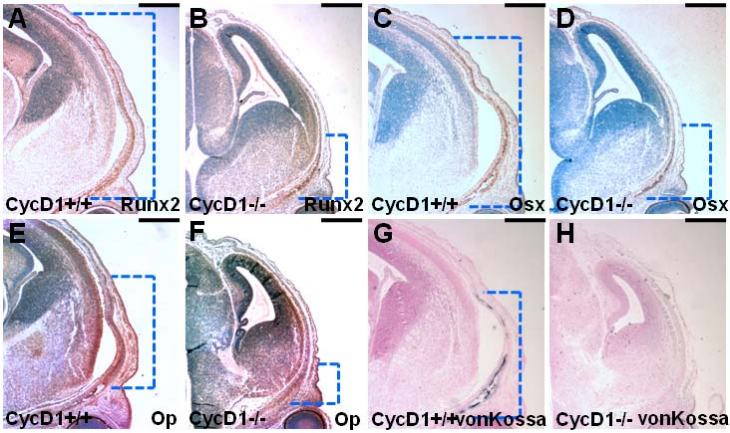
**Cyclin D1 deficiency impairs calvarial development during embryogenesis**. Immunostaining analysis examines the expression of Runx2 (A, B), Osterix (OSX, C, D) and Osteopontin (OP, E, F) in cyclin D1+/+ (A, C, E) and cyclin D1-/- (B, D, F) mice. Von Kossa staining reveals that the deletion of cyclin D1 results in the absence of bone mineralization at the calvaria (Figure 6G, H). The broken lines indicate the positively stained domains. Scale bar, 0.5 mm (A-H).

To elucidate the mechanism underlying the calvarial abnormalities of cyclin D1-/- mice, we examined development of the osteoblast lineage during embryogenesis. At E15.5, the cyclin D1 deletion reduced the calvarial regions, expressing Runx2 (Figure 6A, B), Osterix (Figure 6C, D) and Osteopontin (Figure 6E, F). Similar defects were also observed at E17.5 (see below and data not shown). In the E15.5 mutants, bone mineralization was not yet detected by von Kossa staining analysis (Figure 6G, H), suggesting an impairment of osteoblast development caused by the loss of cyclin D1.

**Figure 7:**
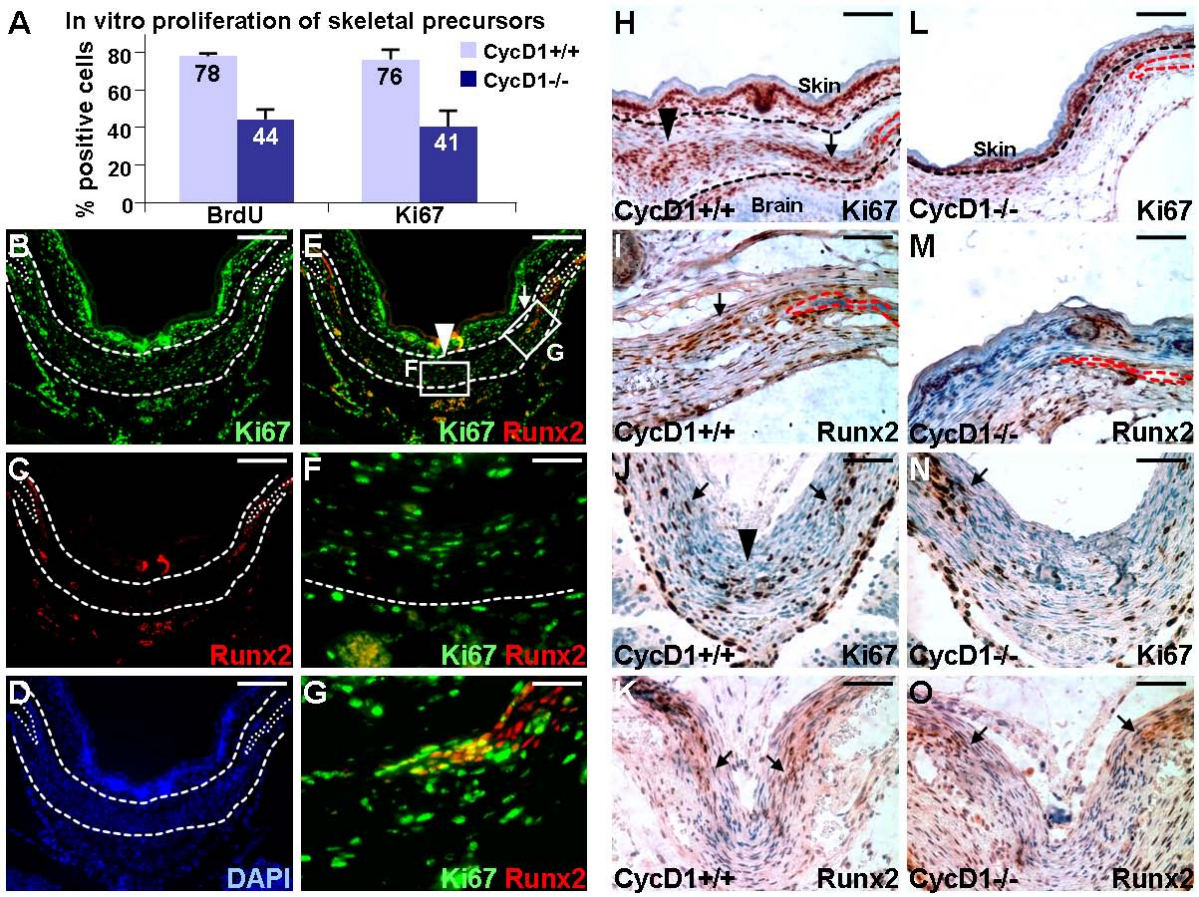
**Expansion of skeletogenic precursors is impaired by the cyclin D1 mutation**. (A) Cyclin D1 mutation reduces skeletal precursor proliferation in vitro. Primary calvarial cells, isolated from the CycD1+/+ and CycD1-/- newborns, are analyzed by BrdU incorporation and Ki67 staining assays. The immunostained images are taken randomly to determine the percentage of positive cells (n = 3). (B-G) Double labeling reveals the proliferating skeletogenic precursor populations that are Runx2 negative and positive in the metopic suture. Co-immunostaining of Ki67 (B) and Runx 2 (C), and counterstaining with DAPI (D), show cells undergoing mitotic divisions with (arrow) or without (arrowhead) the expression of Runx2 (E) at E17.5. Enlargements of the insets (E) are shown in F and G. (H-O) Impaired skeletogenic activity at the osteogenic fronts of CycD1-/- during embryogenesis is restored at birth. Immunostaining of the E17.5 (H, I, L, M), or newborn (J, K, N, O), CycD1+/+ (H-K) and CycD1-/- (L-O) metopic sutures with Ki67 (H, J, L, N) and Runx2 (I, K, M, O) identify cells undergoing mitotic divisions and expressing Runx2, respectively. At E17.5, the deletion of cyclin D1 affects the presence of Ki67 (H, L) and Runx2 (I, M) positive cells at both suture mesenchyme (Runx2-, arrowhead) and osteogenic fronts (Runx2+, arrows). At newborn, the expression of Ki67 (J, N) remains affected at the midline suture (arrowhead), but becomes unaffected at the osteogenic fronts (arrows) of CycD1-/- which also contain Runx2-expressing cells (K, O). The suture areas and osteogenic fronts are highlighted by broken lines. Scale bars, 200 μm (B-E); 100 μm (H, L); 50 μm (F, G, I-K, M-O).

The impaired osteoblast development might be attributed to defects in expansion of the skeletal precursors in cyclin D1-/- mutants. We therefore examined the well established role of cyclin D1 in cell proliferation affected by the mutation. First, in vitro proliferation analysis of skeletal precursors isolated from the wild type and mutant calvaria indicated that the cyclin D1 deletion has a negative effect on cell proliferation (Figure 7A). We next examined the skeletal precursors undergoing mitotic divisions which are positive for Ki67 and Runx2 in the metopic sutures (Figure 7B-D). Co-localization analysis revealed two populations of skeletogenic precursors in the suture mesenchyme (Figure 7E). While the proliferating cells did not express Runx2 at the midline suture region (Figure 7F), they are Runx2 positive at the osteogenic fronts (Figure 7G). The results thus suggest that there are two distinct populations of naïve cells actively proliferating in the developing suture mesenchyme.

Further examination revealed that cyclin D1 deficiency reduced both Runx2 negative and positive populations at E17.5 (Figure 7H, I, L, M). However, at newborn, the expression of Ki67 (Figure 7J, N) remained affected at the suture mesenchyme, but became unaffected at the osteogenic fronts of cyclin D1-/- which are Runx2-expressing cells (Figure 7K, O). This implies that the cyclin D1 ablation interferes with the expansion of naïve cells in the developing suture at early developmental stages. As the development proceeds, the effects on the committed osteoprogenitors were somehow alleviated. At newborn, we also observed the status of FGF and BMP signaling in the skeletal precursors at the osteogenic fronts of cyclin D1-/-, which were comparable to the wild type counterparts (Additional file 1 Figure S1). The skeletogenic activities at the mutant osteogenic fronts appeared to be restored at newborn.

**Figure 8:**
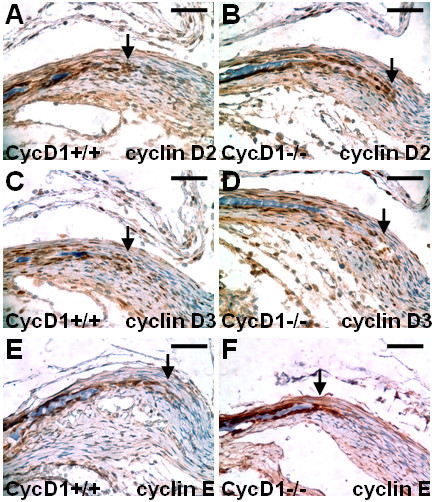
**Presence of other G1/S cyclins at the osteogenic fronts of cyclin D1-/-**. Immunostaining analysis reveals the expression of cyclin D2 (A, B), D3 (C, D) and E (E, F) at the osteogenic fronts of wild type (A, C, E) and cyclin D1-null (B, D, F) metopic suture at newborn. Arrows indicate the osteogenic fronts. Scale bar, 50 μm (A-F).

The lack of sufficient supply of skeletal precursors seems to be the cause of ossification delay which results in a wide open suture between the bone plates at birth. Both the Runx2 negative and positive skeletogenic precursors are affected during embryogenesis. However, the cyclin D1 deletion disrupts only proliferation of the naïve cells which do not express Runx2 while the Runx2 positive cells at the osteogenic fronts maintain active proliferation at newborn. One possibility is that the loss of cyclin D1 is compensated by other D-type cyclins [13]. Alternatively, other cyclins with similar function, e.g. E-type, have been reported to be elevated due to the loss of cyclin D1 [14]. Analysis of mice expressing only a single D-type cyclin each has revealed that the tissue specific expression patterns of the other remaining cyclin D proteins are lost [15]. To test these possibilities, we therefore examined the expression of other G1/S cyclins. The expression of cyclin D2, D3 and E were detected at the osteogenic fronts at newborn (Figure 8A, C, E). Their strong presences were also found in the cyclin D1 mutants (Figure 8B, D, F). The results suggest that compensations by these cyclins with similar function to cyclin D1 could occur at the osteogenic fronts to overcome the mutation. Thus provides a mechanism underlying the independence of cyclin D1 on postnatal development of the calvaria.

## Discussion

Studies in the past have suggested the importance of β-catenin signaling in modulating osteoblast proliferation and differentiation in health and disease [16-23]. It has been previously proposed that proliferation of skeletal precursors requires β-catenin [16,18]. In the subsequent development of osteoblasts, β-catenin needs to be activated [16-18]. Our prior study of mice with disruption of Axin2 also indicates a dual role of β-catenin in expansion of skeletal precursors and their differentiation into osteoblasts [6]. There is no question about the essential function of β-catenin in regulating the expansion of skeletal precursors. However, there is a need to obtain additional in vivo evidence for its involvement in the maturation process. This has been an obstacle due to the effect of cell proliferation also interfering with cell differentiation. Indeed, both deletion and stimulation of β-catenin in the skeletogenic mesenchyme result in delay of calvarial development at embryonic stages [16,17].

Using sophisticated mouse genetic systems, we are able to further our investigation by manipulating the activity of β-catenin in the Axin2-expressing cells within the suture mesenchyme. This study provides direct in vivo evidence to support that β-catenin regulates both proliferation and differentiation of osteogenic precursors. Stimulation of β-catenin with transgenic expression of a dominant mutant protein in the suture mesenchyme results in calvarial deformities. In these mutants, the expansion of skeletal precursors and their differentiation into osteoblasts are highly stimulated. These abnormalities are reminiscent to those observed in the Axin2 mutants. The gain-of-function study strongly supports the hypothesis for a dual role of β-catenin in cell proliferation and differentiation during osteoblast development [6,7].

**Figure 9:**
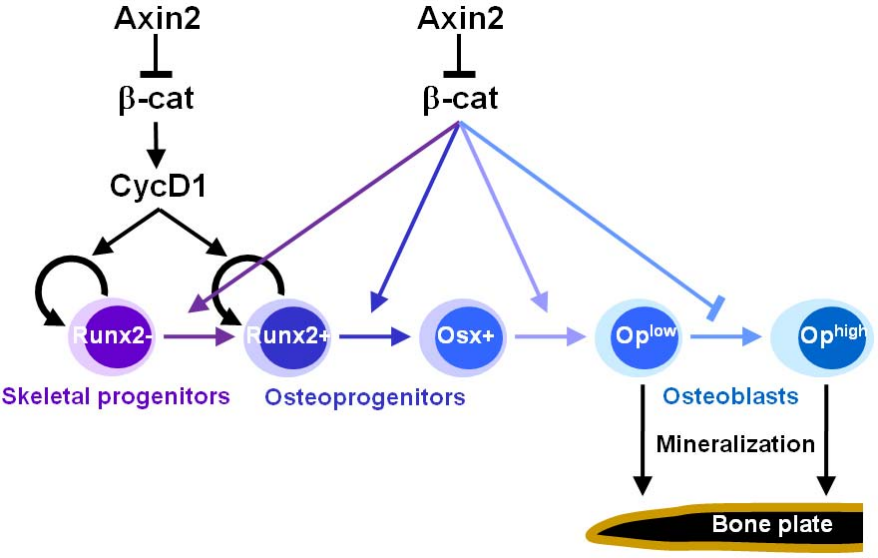
**Model for osteoblast lineage development mediated by the Axin2/β-catenin/cyclin D1 regulatory pathway**. Growing evidence suggests that Axin2/β-catenin regulates the self renewal of mesenchymal stem cells (MSCs) and the expansion of skeletogenic progenitors during calvarial morphogenesis [6-8]. This study shows that cyclin D1 is a downstream modulator, acting in this regulatory axis. Furthermore, canonical Wnt signaling promotes specification of osteoprogenitors which are Runx2+ and Osx+, but prohibits terminal differentiation of mature osteoblasts expressing high levels of Op. Nonetheless, osteoblasts expressing low levels of Op are matrix-secreting cells capable of mediating bone synthesis.

Increasing lines of evidence [6-8] implicate that canonical Wnt signaling is required for self renewal of mesenchymal stem cells and the expansion of skeletogenic precursors during calvarial morphogenesis. This notion is manifest on the sβcat^Ax2 ^model (Figure 9). Furthermore, the current study is in agreement with previous suggestion for a continuing role of β-catenin in specification of osteoprogenitors which are Runx2+ and Osterix+ osteoblasts [16,24]. There has been a report indicating that expression of the sβcat^Ax2 ^mutant prevents terminal differentiation of osteoblasts expressing high levels of Osteocalcin [18]. We obtained similar results showing that stimulated β-catenin signaling inhibits the mature osteoblasts to express high levels of Osteopontin (Figure 9). Nonetheless, this inhibitory effect does not impair bone mineralization to take place. Because enhanced ossification is clearly demonstrated in the sβcat^Ax2 ^skulls, our results support the previous observation that β-catenin is essential for maturation of osteoblast progenitors into bone matrix-secreting osteoblasts [18]. Therefore, osteoblasts expressing low levels of Osteopontin and Osteocalcin are still capable of bone synthesis (Figure 9). Genetic studies also indicate that the deletion of Osteocalcin [25] or Osteopontin [26] in mice does not impair skeletal development.

The sβcat^Ax2 ^mice do not seem to display the exact same phenotype as the Axin2 mutants. Axin2, working upstream of β-catenin in the canonical Wnt pathway, might possess additional functions affecting other signaling molecules not regulated by β-catenin. Alternatively, the presence of *Axin1 *might compensate for the loss of *Axin2 *in a certain degree as these two genes can functionally substitute for each other [27]. While Axin1 is still able to regulate β-catenin in the Axin2 mutants, the stabilized form of β-catenin can no longer be modulated by Axin proteins in the sβcat^Ax2 ^mice resulting in the phenotypic difference between the two models.

Because of our model permitting conditional stimulation of β-catenin, we have been able to overcome the embryonic lethality and examine its role in postnatal development of the craniofacial skeleton. In early juveniles, the calvarium grows rapidly while skeletal precursors are required to expand significantly. Alterations of β-catenin at this stage cause severe proliferation and differentiation defects. In contrast, the skeletal precursor pool is relatively quiescent after mice are 3 weeks old. In late juveniles, expansion of skeletal precursors does not seem to be affected by aberrant β-catenin signaling while high levels of bone ossification are detected (Mirando and Hsu, unpublished). Unfortunately, mice with inducible expression of the dominant β-catenin protein are unable to survive beyond 4 weeks after the initial activation of transgene takes place. The lethality issue, most likely due to the Axin2 promoter being active in other tissues and organs, has prevented us from assessing the long term effect of the transgenic expression. Nonetheless, the new finding nicely complements our previous work using a loss-of-function analysis in which haploid deficiency of β-catenin alleviates the skull defects caused by the Axin2 disruption [7].

We have previously shown that as a transcriptional target of Wnt, cyclin D1 is modulated by alterations of the Axin2/β-catenin regulatory pathway [6,7]. However, there is a lack of information on linking the cyclin D1 function to skeletogenesis [28,29]. To assess the role of cyclin D1 in calvarial morphogenesis, a genetic study was performed. Cyclin D1 apparently has an essential function in calvarial development at embryonic stages. In the cyclin D1 knockout embryos, the expansion of both Runx2 negative and positive skeletal precursor populations are impaired at the suture mesenchyme and osteogenic fronts, respectively. Deficiencies in cell proliferation and differentiation have been shown to cause ossification delay of the skull [30,31]. We have identified similar types of ossification deficiencies in the cyclin D1 knockouts. The finding thus provides first genetic evidence for the involvement of cyclin D1 in calvarial morphogenesis (Figure 9).

As a direct transcriptional target of β-catenin, cyclin D1 acts in this regulatory pathway. However, the loss of cyclin D1 interferes with only the Runx2 negative naïve cells within the suture mesenchyme, but not the Runx2 positive progenitors at the osteogenic fronts at newborns. The skeletogenic signaling pathways of FGF and BMP are also unaffected at the osteogenic fronts. Our data imply that the restoration of skeletogenic activities at the osteogenic fronts of cyclin D1-/- might be a compensation effect of other cyclins with similar function. The loss of cyclin D1 may be compensated by cyclin D2, D3 and E. Nevertheless, we cannot rule out the possibility that the lethality associated with the cyclin D1 deletion could result in selected phenotypic representation. This is because only 72% of mice with disruption of cyclin D1 able to survive after birth [28]. The mutants with most severe phenotypes are unable to be obtained due to abnormalities in other tissues and organs. Furthermore, our study of cyclin D1 in craniofacial skeletogenesis raises a question on its involvement in development of the axial and appendicular skeletons. It remains to be determined whether the D-type cyclin(s) has a role in other developments and maintenance of the body skeleton.

## Conclusions

Using sophisticated mouse genetic studies, we demonstrate that aberrant stimulation of β-catenin causes calvarial defects resembling those of the Axin2 mutation. Constitutive activation of β-catenin enhances both expansion of skeletogenic precursors and bone ossification. Together with our previous findings, these results indicate that β-catenin plays an essential role in the calvarial morphogenetic signaling pathways. In addition, as a direct transcriptional target of β-catenin, cyclin D1 is required for the expansion of skeletal precursors during calvarial morphogenesis. This study provides genetic evidence to further support the importance of Wnt signaling in calvarial morphogenesis, suggesting a key regulatory pathway of Axin2/β-catenin/cyclin D1 in development of the suture mesenchyme.

## Methods

### Mouse strains

The Axin2-rtTA [32,33], TRE-lacZ [34,35], TRE-H2BGFP [8,33,34], TRE-Cre [8], β-catΔEx3Fx [9], cyclin D1 [28] mouse strains and genotyping methods were reported previously. To test the Dox-inducible expression system, mice carrying the Axin2-rtTA and TRE-lacZ transgenes were obtained and treated with Dox (2 mg/ml plus 50 mg/ml sucrose) for several days as described [8,32,34,35]. For generating the sβcat^Ax2 ^mouse strain, mice carrying Axin2-rtTA and TRE-Cre transgenes were first bred into the β-catΔEx3Fx heterozygous background [8]. The expression of a degradation-deficient β-catenin in the Axin2-expressing cells was then induced by Dox treatment at E16.5 or E17.5. Care and use of experimental animals described in this work comply with guidelines and policies of the University Committee on Animal Resources at the University of Rochester.

### Cells

Isolation and culture of primary skeletal precursors were performed as described [6]. Briefly, calvarial cells isolated from newborns were cultured in αMEM media containing 10% fetal calf serum. Only the first passage cells were used for the study. To induce the expression of the transgene, Dox was added to the culture media for 8 hours. Cells (1 × 10^4^) were seeded in 24 well plates, and maintained in differentiation media containing 50 μg/ml ascorbic acid and 4 mM β-glycerophosphate for 6 days, followed by alkaline phosphatase staining as described [6].

### Histology, skeletal preparation, β-gal staining and GFP analysis

Skulls were fixed for skeletal preparation or paraffin embedded, sectioned and stained with hematoxylin/eosin for histology or von Kossa staining as described [6,7]. Details for β-gal staining in whole mounts or sections were reported previously [32,34,35]. For analysis in sections, newborn skulls were further fixed in 4% PFA/PBS, processed for frozen section, and evaluated by histology using Zeiss Axio Observer microscope (Carl Zeiss, Thornwood, NY).

### Immunostaining analysis

Immunological staining with avidin:biotinlylated enzyme complex was performed as described [6,7]. The staining was visualized by enzymatic color reaction or fluorescence according to the manufacture's specification (Vector Laboratories, Burlingame, CA). Images were taken using Zeiss Axio Observer microscope. Mouse monoclonal antibodies Cdk4 (Cell Signaling, Danvers, MA), BrdU (Thermo Fisher, Fremont, CA), Runx2 (MBL International, Woburn, MA), Osteopontin (Developmental Studies Hybridoma Bank, Iowa City, IA), cyclin D2 (Thermo Fisher), cyclin D3 (Thermo Fisher), cyclin E (Santa Cruz) and ABC (recognizing activated/non phosphorylated form of β-catenin, Upstate; Billerica, MA); rabbit polyclonal antibodies cyclin D1 (Thermo Fisher), Fgfr1 (Santa Cruz, Santa Cruz, CA), Fgfr2 (Santa Cruz), Fgf2 (Santa Cruz), Ki67 (Thermo Fisher), Osterix (Abcam, Cambridge, MA) and phospho-Smad1/5/8 (Cell Signaling) were used in these analyses. For statistical evaluation, the stained images were taken to determine the percentage of positive cells by counting the positively stained cells in a total population of cells (positive plus negative). The counting process was repeated in different samples for a number of times (n represents the number of animals or cell cultures) to obtain the mean values and error bars. The *p *values were obtained using student *t *test.

## Authors' contributions

AJM, HIY and WH established the mouse models and carried out the genetic studies. AJM, TM and WH designed the experiments. AJM, TM, JF and HIY performed molecular characterizations, cell proliferation and differentiation assays and statistical analyses. AJM, TM and WH wrote the paper. All authors read and approved the final manuscript.

## Supplementary Material

Additional file 1 **Figure S1: The skeletogenic activities are restored at the osteogenic fronts of cyclin D1-/- after birth.** Immunostaining analysis reveals that the expression of Fgf2 (A, B), Fgfr1 (C, D), Fgfr2 (E, F) and phosphorylated Smad1/5/8 (G, H) is not affected in the mutant metopic sutures (B, D, F, H) compared to the control (A, C, E, G) at newborn. Arrows indicate the osteogenic fronts. Scale bars, 50 μm (A-H). Click here for file
