# Supplementary figures and images for: β-catenin/cyclin D1 mediated development of suture mesenchyme in calvarial morphogenesis
Source: BMC Dev Biol. 2010 Nov 26;10:116. doi: 10.1186/1471-213X-10-116 (PMC3001432; doi:10.1186/1471-213X-10-116)

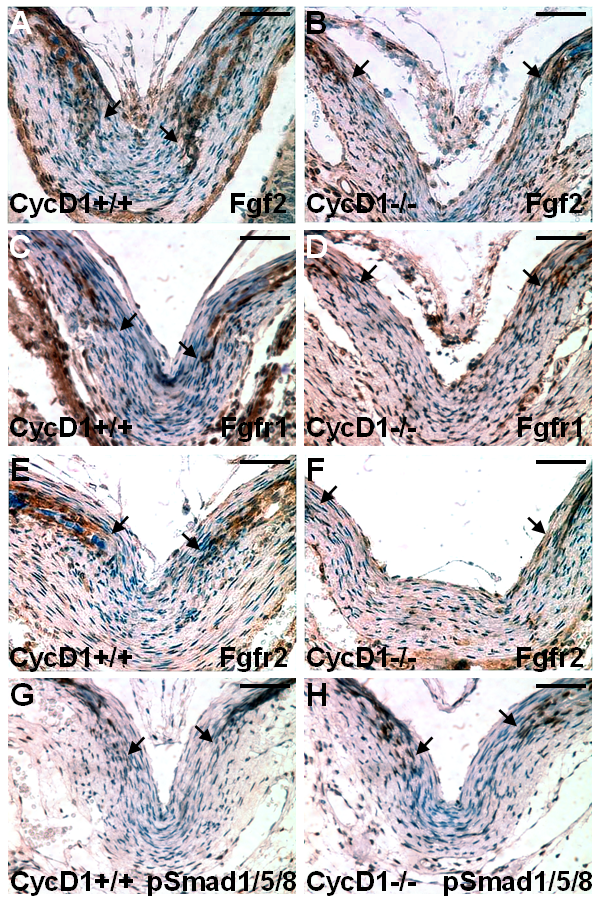

Supplement: Additional file 1 — Figure S1: The skeletogenic activities are restored at the osteogenic fronts of cyclin D1-/- after birth. Immunostaining analysis reveals that the expression of Fgf2 (A, B), Fgfr1 (C, D), Fgfr2 (E, F) and phosphorylated Smad1/5/8 (G, H) is not affected in the mutant metopic sutures (B, D, F, H) compared to the control (A, C, E, G) at newborn. Arrows indicate the osteogenic fronts. Scale bars, 50 μm (A-H). [file 1471-213X-10-116-S1.TIFF]
